# Racial and Ethnic Differences in Internal Medicine Residency Assessments

**DOI:** 10.1001/jamanetworkopen.2022.47649

**Published:** 2022-12-29

**Authors:** Dowin Boatright, Nientara Anderson, Jung G. Kim, Eric S. Holmboe, William A. McDade, Tonya Fancher, Cary P. Gross, Sarwat Chaudhry, Mytien Nguyen, Max Jordan Nguemeni Tiako, Eve Colson, Yunshan Xu, Fangyong Li, James D. Dziura, Somnath Saha

**Affiliations:** 1 Department of Emergency Medicine, New York University Grossman School of Medicine, New York, New York; 2 Department of Psychiatry, Yale School of Medicine, New Haven, Connecticut; 3 Kaiser Permanente Bernard J. Tyson School of Medicine, Pasadena, California; 4 Accreditation Council for Graduate Medical Education, Chicago, Illinois; 5 Department of Internal Medicine and Office of Workforce Innovation and Community Engagement, University of California, Davis; 6 Section of General Internal Medicine, Yale School of Medicine, New Haven, Connecticut; 7 MD-PhD Program, Yale School of Medicine, New Haven, Connecticut; 8 Department of Medicine, Brigham and Women’s Hospital, Boston, Massachusetts; 9 Department of Pediatrics, Washington University School of Medicine, St Louis, Missouri; 10 Yale Center for Analytic Sciences, Yale School of Public Health, New Haven, Connecticut; 11 Section of General Internal Medicine, Johns Hopkins University School of Medicine, Baltimore, Maryland

## Abstract

**Importance:**

Previous studies have demonstrated racial and ethnic inequities in medical student assessments, awards, and faculty promotions at academic medical centers. Few data exist about similar racial and ethnic disparities at the level of graduate medical education.

**Objective:**

To examine the association between race and ethnicity and performance assessments among a national cohort of internal medicine residents.

**Design, Setting, and Participants:**

This retrospective cohort study evaluated assessments of performance for 9026 internal medicine residents from the graduating classes of 2016 and 2017 at Accreditation Council of Graduate Medical Education (ACGME)–accredited internal medicine residency programs in the US. Analyses were conducted between July 1, 2020, and June 31, 2022.

**Main Outcomes and Measures:**

The primary outcome was midyear and year-end total ACGME Milestone scores for underrepresented in medicine (URiM [Hispanic only; non-Hispanic American Indian, Alaska Native, or Native Hawaiian/Pacific Islander only; or non-Hispanic Black/African American]) and Asian residents compared with White residents as determined by their Clinical Competency Committees and residency program directors. Differences in scores between Asian and URiM residents compared with White residents were also compared for each of the 6 competency domains as supportive outcomes.

**Results:**

The study cohort included 9026 residents from 305 internal medicine residency programs. Of these residents, 3994 (44.2%) were female, 3258 (36.1%) were Asian, 1216 (13.5%) were URiM, and 4552 (50.4%) were White. In the fully adjusted model, no difference was found in the initial midyear total Milestone scores between URiM and White residents, but there was a difference between Asian and White residents, which favored White residents (mean [SD] difference in scores for Asian residents: −1.27 [0.38]; *P* < .001). In the second year of training, White residents received increasingly higher scores relative to URiM and Asian residents. These racial disparities peaked in postgraduate year (PGY) 2 (mean [SD] difference in scores for URiM residents, −2.54 [0.38]; *P* < .001; mean [SD] difference in scores for Asian residents, −1.9 [0.27]; *P* < .001). By the final year 3 assessment, the gap between White and Asian and URiM residents’ scores narrowed, and no racial or ethnic differences were found. Trends in racial and ethnic differences among the 6 competency domains mirrored total Milestone scores, with differences peaking in PGY2 and then decreasing in PGY3 such that parity in assessment was reached in all competency domains by the end of training.

**Conclusions and Relevance:**

In this cohort study, URiM and Asian internal medicine residents received lower ratings on performance assessments than their White peers during the first and second years of training, which may reflect racial bias in assessment. This disparity in assessment may limit opportunities for physicians from minoritized racial and ethnic groups and hinder physician workforce diversity.

## Key Points

**Question:**

Are there disparities in the assessment of internal medicine residents associated with race and ethnicity?

**Findings:**

In this cross-sectional study of 9026 internal medicine residents, Asian residents and residents historically underrepresented in medicine by race and ethnicity received lower ratings on assessments than their White peers during the first and second years of training. These differences abated by the final assessment in year 3 of training.

**Meaning:**

These findings suggest that internal medicine residents from minoritized racial and ethnic groups may experience bias in assessment; these disparities in assessment may limit future career opportunities for residents from these groups and hinder workforce diversity.

## Teaser

This cross-sectional study examines whether race and ethnicity are associated with performance evaluation ratings of internal medicine residents.

## Introduction

The National Academy of Medicine has long recommended increasing diversity in the health care workforce as a crucial intervention to reduce racial health disparities.^1,2^ Nevertheless, Black, Hispanic, and American Indian and Alaska Native physicians remain underrepresented in medicine (URiM). Although Asian physicians are not underrepresented, Asian medical students are less likely to be selected for prestigious honor societies, and as they progress in their careers, Asian faculty members are less likely to hold departmental leadership positions.^3,4,5,6,7^ While prior efforts to increase diversity have focused on recruitment,^8,9,10,11^ there remains a need to identify structural barriers within the learning environment that hinder workforce diversity. One aspect of this challenge is evaluating whether there is racial bias in graduate medical education (GME) assessments.

Assessments of GME trainees inform important decisions regarding promotion, chief resident selection, readiness for unsupervised practice, and entry into competitive subspecialty GME programs. A previous study^12^ found that even small differences in assessment can accumulate longitudinally and prevent career advancement. Consequently, bias in assessments may limit career opportunities for physicians from minoritized racial and ethnic groups in community practice and academic medicine.

Assessments in internal medicine (IM) are especially impactful because of the field’s contribution to the physician workforce. Nearly one-third of adult primary care physicians complete an IM residency.^13^ Internal medicine residency is also a prerequisite for most adult subspecialties, including cardiology, hematology-oncology, pulmonary and critical care, and gastroenterology—fields where Black, Hispanic, and American Indian and Alaska Native physicians remain underrepresented.^14^

In 2013, the Accreditation Council of Graduate Medical Education (ACGME) launched a new assessment system of competency-based clinical milestones.^15,16^ The Milestone system is nationally standardized and allows longitudinal assessment of resident performance across 6 domains of competency. The Milestone system was designed to support formative assessment, bolster professional development, and enhance the quality of assessments.^15,16^

However, a recent study^17^ of ACGME’s Milestone assessment system reported differences in assessment by race and ethnicity, suggesting that the Milestone assessment system may be vulnerable to bias. This study^17^ was limited in cohort size and the number of included GME training sites; therefore, it is unclear whether these findings are generalizable. To address this important knowledge gap, we examined Milestone ratings across all clinical competency domains for racial and ethnic differences among a national cohort of IM residents.

## Methods

### Study Setting and Participants

We conducted a retrospective cohort study of ACGME Milestone assessments of IM residents from the graduating classes of 2016 and 2017. The initial cohort included all IM residency programs (N = 488) and 16 902 residents. We excluded residents (n = 1726 [10.2%]) from programs (n = 94 [19.2%]) with incomplete GME tracking data during the study period, and we excluded residents (n = 2220 [13.1%]) from programs (n = 89 [18.2%]) that did not have at least 1 URiM, 1 Asian, and 1 White resident during the study period to ensure that racial and ethnic differences in assessment could be analyzed at each residency program. Four residents were excluded because they did not have Milestones data reported to the ACGME. All data were deidentified. This study followed the Strengthening the Reporting of Observational Studies in Epidemiology (STROBE) reporting guideline and was deemed exempt from the need for informed consent by the Yale Institutional Review Board.

### ACGME Milestone Data

The ACGME Milestones are used by residency programs’ Clinical Competency Committees (CCCs) to assess resident knowledge, skills, attitudes, and other attributes for each of the 6 clinical competency domains: medical knowledge, patient care, interpersonal and communication skills, practice-based learning and improvement, professionalism, and systems-based practice. Each of these competency domains also has subcompetencies, totaling 22 subcompetencies.^18^ Each subcompetency is rated on 5 levels of performance that are described in narrative terms. Residents may be rated at in-between levels (transition zones), resulting in a 9-point scale.

A CCC at the level of the residency determines Milestone ratings for each resident by synthesizing data from numerous sources, including resident assessments by faculty and peers, direct observation, and in-service examination scores. The CCC provides these scores to the residency program director, who has the ultimate authority to assign Milestone developmental scores. Residency program directors report these data to the ACGME twice a year, totaling 6 performance assessments per resident.

### Race and Ethnicity

Race and ethnicity were included in 7 categories: Hispanic; non-Hispanic American Indian, Alaska Native, or Native Hawaiian/Pacific Islander; non-Hispanic Asian; non-Hispanic Black/African American; non-Hispanic White; and unknown or other (analyses on Hispanic and other racial subgroups were not conducted). Resident race and ethnicity data came from the Association of American Medical Colleges’ (AAMC’s) data services and applications. While the most recent self-reported data were prioritized, some race and ethnicity data came from AAMC data sources where the resident did not self-report (eg, the GME Track). Residents who reported race and ethnicity in 2 or more groups were categorized as multiracial.

For this study, URiM referred to residents who identified as Hispanic only; non-Hispanic American Indian, Alaska Native, or Native Hawaiian/Pacific Islander only; or non-Hispanic Black/African American. For analytic purposes, multiracial residents self-reporting at least 1 race and ethnicity considered URiM were categorized as URiM, and residents self-reporting their race and ethnicity to be Asian and White were categorized as Asian.

Study investigators received resident race and ethnicity data from the AAMC and resident Milestones data from the ACGME. Investigators linked these data sets using unique identifiers generated for each resident. We excluded residents who were not US citizens because their race and ethnicity data were not available to the study team (n = 3651 [21.6%]). We also excluded 275 residents (1.6%) who did not self-report their race or ethnicity.

### Study Outcomes

The primary outcome was midyear and year-end total Milestone scores. The total Milestone score is the sum of all scores in all 6 competency domains. We also reported scores for each of the 6 core competencies. Finally, we included 2 outcomes, which represented assessments at the extremes of the assessment scale. That is, we identified the frequency in which residents were deemed ready for unsupervised practice (mean Milestone score of ≥7) and, conversely, whether a resident received a critical deficiency (Milestone score of 0 for any subcompetency). Differences in the assessment of readiness for unsupervised practice or the receipt of a critical deficiency could have implications for future learning opportunities, graduation, and employment.

### Statistical Analysis

Analyses were conducted between July 1, 2020, and June 31, 2022. We summarized the residents’ characteristics by 3 race and ethnicity groups (URiM, Asian, and White) using descriptive statistics, including mean (SD) for continuous variables and number (percentage) for categorical variables. We used analysis of variance or the χ^2^ test for group comparisons as appropriate.

We conducted a multilevel, mixed-effects linear regression to examine the association between race and ethnicity and Milestone competency scores. We used an unstructured covariance matrix to accommodate within-participant correlation from repeated assessments for each resident. To account for clustering, residents were nested within training programs, using a random effect for programs. Our model included fixed effects for residency year, resident race and ethnicity, and their interaction. We adjusted for sex, age, and United States Medical Licensing Examination (USMLE) Step 2 scores, which represent a proxy for baseline medical knowledge. We used linear contrast to compare Milestone scores by postgraduate year (PGY) and group. Least squares means and 95% CIs were reported. All available observations were used in the mixed-effects modeling without excluding residents who had missed assessments at certain time points. A mixed-model approach is robust to missing data, assuming a missing-at-random mechanism.

We assessed the likelihood of a resident being rated as ready for unsupervised practice for each core competency, as well as the odds of a resident receiving a critical deficiency for any subcompetency using logistic regression accounting for resident sex, age, and USMLE Step 2 scores and clustering within training programs. We explored the likelihood of a resident being rated ready for unsupervised practice at the midyear assessment and end-of-year assessment in PGY3. We also evaluated the odds of a resident receiving a critical deficiency at each of the semiannual assessments.

We performed a secondary, exploratory analysis using the same approach described above for a subset of residents completing IM training at historically black colleges and universities (HBCUs): Meharry Medical College, Morehouse School of Medicine, and Howard University College of Medicine. Compared with predominantly White institutions, HBCUs often have greater racial and ethnic diversity among trainees and faculty. We posited that this increased diversity and interracial contact among faculty and residents might mitigate bias in evaluations.^19,20,21,22^

We performed analyses using SAS software, version 9.4 (SAS Institute Inc). Statistical significance was presumed at *P* < .05 (2-tailed test) for all analyses.

## Results

### Internal Medicine Resident Characteristics

Our final study cohort included 9026 residents (5032 male [55.8%]; 1216 [13.5%] URiM; 3258 [36.1%] Asian; 4552 [50.4%] White) from 305 IM residency programs. The mean (SD) USMLE Step 2 score was 239.8 (17.2) (Table 1).

**Table 1.** Demographic Characteristics of the Internal Medicine Residents

Characteristic	Finding^a^ (N = 9026)
Sex	
Male	5032 (55.8)
Female	3994 (44.2)
Race and ethnicity^b^	
Asian	3258 (36.1)
Asian only	3129 (34.7)
Asian and White	129 (1.4)
URiM	1216 (13.5)
URiM only	998 (11.1)
URiM-multiracial	218 (2.4)
White	4552 (50.4)
USMLE Step 2 CK scores	
Mean (SD)	239.8 (17.2)
Median (range)	241.0 (163.0-285.0)

Abbreviations: CK, content knowledge; URiM, underrepresented in medicine; USMLE, United States Medical Licensing Examination.

^a^ Data are presented as number (percentage) of residents unless otherwise indicated.

^b^ Asian indicates any residents who reported their race or ethnicity to be Asian, which could include Asian and White. Asian only indicates residents who marked only Asian as their race or ethnicity and not any other races or ethnicities.

### Milestone Ratings

**Figure 1.**
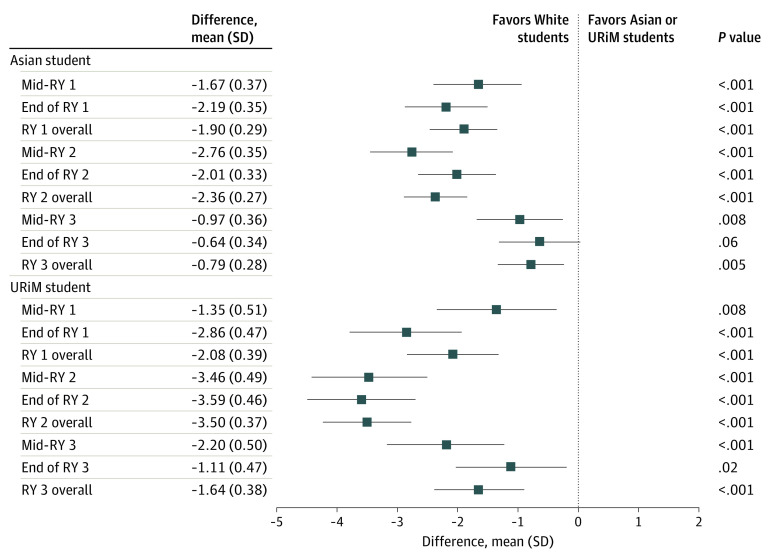
Unadjusted Total Milestone Scores Underrepresented in medicine (URiM) refers to residents who identified as Hispanic only; non-Hispanic American Indian, Alaska Native, or Native Hawaiian/Pacific Islander only; or non-Hispanic Black/African American. RY indicates residency year. White residents are the reference group for calculating the difference in Milestone scores.

In our unadjusted model examining the observed and reported total Milestone scores, URiM and Asian residents received lower total scores than White residents on the initial PGY1 midyear assessments (mean [SD] difference in scores for URiM residents, −1.35 [0.51], *P* = .008; mean [SD] difference in scores for Asian residents, −1.67 [0.37]; *P* < .001). The mean difference between URiM and Asian residents’ total scores compared with White residents increased during PGY2 (mean [SD] difference in scores for URiM residents, −3.5 [0.37]; *P* < .001; mean [SD] difference in scores for Asian residents, −2.36 [0.27]; *P* < .001). Racial and ethnic differences in assessment began to decrease in PGY3, and by the PGY3 year-end assessment, there was no statistically significant difference in total Milestone scores between Asian and White residents; however, a statistically significant difference between URiM and White residents remained, favoring White residents (mean [SD] difference in scores for URiM residents, −1.11 [0.47]; *P* = .02) (Figure 1).

A similar pattern of racial and ethnic differences in Milestones scores was present in each of the 6 competency domains in the unadjusted model. Asian and URiM residents received lower Milestone scores than their White peers in all competency domains at the PGY1 year-end assessment, and these differences increased in PGY2 (eFigure 1 in Supplement 1). The mean (SD) difference in assessment in PGY2 between Asian and White residents by competency domain ranged from −0.28 (0.03) for the medical knowledge competency to −0.54 (0.07) for patient care; for URiM residents, this difference in assessment in PGY2 ranged from −0.35 (0.06) for interpersonal and communication skills to −0.92 (0.09) for patient care (*P* < .001 for all). At the PGY3 year-end assessment, parity between Asian and White residents was reached in 4 (medical knowledge, practice-based learning and improvement, professionalism, and interpersonal and communication skills) of 6 competency domains; for URiM residents, parity with White residents was reached in 3 (systems-based practice, professionalism, and interpersonal and communication skills) of 6 domains.

**Figure 2.**
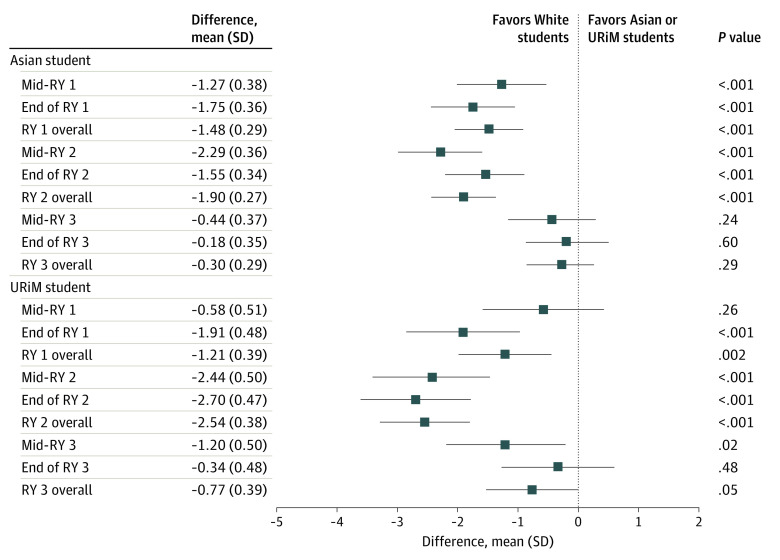
Adjusted Total Milestone Scores Underrepresented in medicine (URiM) refers to residents who identified as Hispanic only; non-Hispanic American Indian, Alaska Native, Native Hawaiian/Pacific Islander only; or non-Hispanic Black/African American. RY indicates residency year. White residents are the reference group for calculating the difference in Milestone scores.

In the fully adjusted model, we found no difference in the PGY1 midyear total Milestone scores between URiM and White residents, but there was a difference between Asian and White residents that favored White residents (mean [SD] difference in scores for Asian residents, −1.27 [0.38]; *P* < .001). However, White residents began to receive increasingly higher scores compared with URiM and Asian residents in subsequent assessments. These disparities peaked in PGY2 (mean [SD] adjusted difference in URiM residents, −2.54 [0.38]; *P* < .001; mean [SD] adjusted difference in Asian residents, −1.9 [0.27]; *P* < .001) (Figure 2). By the PGY3 year-end assessment, the gap between White and Asian and URiM residents’ scores narrowed, and no racial and ethnic difference was found in the total Milestone scores. Trends in racial and ethnic differences among the 6 competency domains mirrored total Milestone scores, with differences peaking in PGY2 and then decreasing in PGY3 such that parity in assessment was reached in all competency domains (eFigure 2A-F in Supplement 1).

### Readiness for Unsupervised Practice

At the PGY3 midyear assessment, Asian residents were 20% to 25% less likely than White residents to be considered ready for unsupervised practice in all Milestone competency domains (adjusted odds ratio, 0.78; 95% CI, 0.69-0.88) (Table 2). URiM residents were almost 15% less likely than White residents to be rated as ready for unsupervised practice in 4 of 6 competency domains (medical knowledge, practice-based learning and improvement, professionalism, and interpersonal and communication skills) at the PGY3 midyear assessment (Table 2). By the PGY3 year-end assessment, Asian residents remained nearly 17% less likely than White residents to be deemed ready for independent practice in interpersonal and communication skills (adjusted odds ratio, 0.83; 95% CI, 0.7-0.99); there were no statistically significant differences in readiness for unsupervised practice in any competency domain between URiM and White residents at the PGY3 year-end assessment.

**Table 2.** Likelihood of Postgraduate Year 3 Residents Being Rated Ready for Independent Practice

Group	OR (95% CI)						
	Overall	Patient care	Medical knowledge	Systems-based practice	Practice-based learning and improvement	Professionalism	Interpersonal and communications skills
Midyear							
Asian	0.78 (0.69-0.88)	0.76 (0.68-0.84)	0.75 (0.68-0.83)	0.76 (0.68-0.84)	0.79 (0.72-0.88)	0.77 (0.70-0.85)	0.79 (0.71-0.87)
URiM	0.92 (0.78-1.09)	0.86 (0.75-1.00)	0.79 (0.68-0.91)	0.89 (0.77-1.02)	0.83 (0.72-0.96)	0.85 (0.74-0.98)	0.83 (0.72-0.95)
White	1 [Reference]	1 [Reference]	1 [Reference]	1 [Reference]	1 [Reference]	1 [Reference]	1 [Reference]
End of year							
Asian	0.95 (0.85-1.06)	0.92 (0.80-1.05)	0.91 (0.78-1.06)	0.88 (0.76-1.02)	0.93 (0.81-1.07)	0.92 (0.79-1.08)	0.83 (0.70-0.99)
URiM	1.05 (0.9-1.22)	1.02 (0.85-1.22)	0.85 (0.7-1.03)	0.90 (0.74-1.10)	0.89 (0.74-1.07)	1.01 (0.81-1.25)	0.89 (0.7-1.12)
White	1 [Reference]	1 [Reference]	1 [Reference]	1 [Reference]	1 [Reference]	1 [Reference]	1 [Reference]

Abbreviations: OR, odd ratio; URiM, underrepresented in medicine.

### Critical Deficiencies

There was no statistically significant difference in the likelihood of a resident receiving a critical deficiency by race and ethnicity. A total of 3306 residents (36.6%) received a critical deficiency during residency. Most critical deficiencies occurred in the first year of residency, and the number of residents receiving a critical deficiency decreased with each successive year (3025 [33.5%] in year 1, 1467 [16.2%] in year 2, and 844 [9.4%] in year 3).

### Historically Black Medical Schools

We identified 73 residents who completed training at HBCU GME programs. Of these residents, 56 (76.7%) were URiM, 11 (15.1%) were Asian, and 6 (8.2%) were White. In our fully adjusted model, we found no significant racial and ethnic differences in total Milestone score and no differences in the ratings for any competency domain during residency. Furthermore, we found no racial and ethnic differences in the likelihood that a resident was deemed ready for unsupervised practice or in the odds of receiving a critical deficiency.

## Discussion

We found that Asian and URiM residents were rated similarly to White residents in the first year of residency; however, racial and ethnic differences in assessment favoring White residents emerged and peaked during PGY2. These differences abated by the final assessment in PGY3.

These results build on prior evidence showing differences in IM performance evaluations between URiM and non-URiM residents at 6 residency programs.^17^ Our findings advance this work by demonstrating racial inequities in Milestone assessments for all trainees from racial and ethnic groups that have been historically marginalized in medicine, including Asian residents. In addition, our study furthers this research by examining assessments in a sample of more than 9000 trainees from 2 successive cohorts of IM residents.

The consistently similar patterns of lower scores in all 6 competency domains for URiM and Asian residents compared with their White peers during the first 2 years of training raise concerns for a global devaluation of resident physicians from minoritized racial and ethnic groups, suggesting the possibility of racial discrimination against trainees from these groups. Although prior literature has demonstrated racial and ethnic disparities in the recognition of academic achievement among medical faculty^5,23,24^ and students,^3,4,7,25^ this study describes similar phenomena among a national cohort of resident physicians.

The racial and ethnic disparities in Milestone scores found in our study are disquieting. Rater bias in assessment has been associated with the development of stereotype threat, mistrust, and disengagement among learners.^26,27,28^ Our finding that the greatest difference in Milestone scores between White residents and trainees from minoritized racial and ethnic groups occurs in PGY2 is salient and warrants investigation in future studies. Resident assessments during PGY2 can influence future career opportunities, including chief resident appointments, job opportunities in academic medicine and community practice, and selection into competitive medical subspecialties.^29^

Although differences in ratings between Asian and White as well as URiM and White residents in competency domains were small, these inequalities could reflect substantive differences in how residents from minoritized racial and ethnic groups are perceived in summative assessments. This possibility is supported by the finding that Asian residents were 20% to 25% less likely than White residents to be rated ready for unsupervised practice in all competency domains just 6 months before the end of residency; URiM residents were almost 15% less likely than White residents to be rated ready for unsupervised practice in 4 of 6 competency domains during the same timeframe.

A notable finding is that racial and ethnic differences in assessment narrow during the third and last year of residency training. The underlying reason for this observation requires additional investigation. One possible explanation is that for residency programs to justify residents’ graduating, programs may feel compelled to deem those residents ready for unsupervised practice. This pressure, whether conscious or subconscious, may mitigate rater bias and thereby reduce racial and ethnic disparities observed in earlier assessments.

Our finding of no statistically significant racial and ethnic differences in Milestone ratings at the HBCU GME programs should be explored further. It is possible that our cohort of residents at HBCUs was not large enough to detect a significant racial and ethnic difference in assessment. Nevertheless, prior work has suggested that a more diverse faculty may decrease people’s implicit biases through positive social contact,^22,30,31,32,33^ and literature from other educational contexts demonstrates that more diverse faculty may decrease racial bias in assessments.^20,21,34^

### Implications

To address the differential assessment by race and ethnicity found in this study, we offer several recommendations. First, IM residency programs could intensify efforts to recruit, develop, and retain racially and ethnically diverse physicians. It is striking that 18.2% of IM residency programs were excluded from our study because of not having even 1 Asian and URiM resident across 2 training classes. Currently, URiM physicians comprise less than 10% of IM faculty^35^ and less than 4% of full professors in academic medicine.^36^ Greater diversity among both residents and faculty could represent an effective intervention to reduce inequity in program assessment and implicit bias.^22,30,33^

Second, IM training programs could conduct routine internal investigations of disparities in assessment as part of their quality improvement efforts.^37^ These data could be examined by the ACGME during accreditation reviews because a diverse, equitable, and inclusive learning environment free of discrimination represents a core component of the ACGME’s Common Program Requirements. Linking equity in assessment to accreditation could represent a powerful incentive to promote equity and inclusion in training.^11,38,39^

Third, because we found the greatest racial and ethnic differences in assessment in PGY2, a formative period of resident development, IM programs may benefit from evaluating additional outcomes potentially influenced by racial and ethnic inequities in assessment. These outcomes include disparities in resident attrition, on-time graduation, awards, fellowship matching, and chief resident selection.

Fourth, the Milestones instrument could be examined for potential inherent susceptibility to bias. Prior research suggests that increased clarity and specificity in grading criteria can mitigate rater bias.^40^ In addition, the numeric scale used to demarcate Milestones may provide evaluators with undue discretion, rendering the tool vulnerable to rater bias.^41^ Mastery grading systems, in which mastery standards are defined and assessments are made along a binary scale (mastered vs not mastered), may alleviate some of the racial and ethnic disparities in assessment described in this study.^42,43^ This recommendation is supported by our finding that Asian and URiM residents were no more likely to receive a critical deficiency than White residents.

### Limitations

Our study has limitations. It is possible that unaccounted factors at the resident and program level could have influenced the observed differences in assessment present in this study. The race and ethnicity of the assessors was unknown, and the degree of concordance between assessors and trainees could affect equity in assessment.^44^ In addition, non-US citizens were excluded from analysis because their race and ethnicity were unknown to the investigative team. Nevertheless, discrimination against physicians who are non-US citizens is well documented.^45,46,47^ Future studies should examine GME assessments by citizenship. The ACGME introduced the Milestones 2.0 assessment system in July 2021. Although the Milestones 2.0 system was not designed with an equity or inclusion lens,^48^ future studies should examine assessments for racial and ethnic disparities in this new system. Our fully adjusted model included USMLE Step 2 scores as an adjustment variable. Several studies^49,50,51^ have demonstrated racial and ethnic differences in standardized tests, including the USMLE. Because of these disparities, it is possible that including USMLE Step 2 scores biases results in our fully adjusted model to the null hypothesis.

In 2019, the ACGME released its first diversity accreditation standard, and it is unknown how this accreditation standard may have influenced racial disparities in assessment. However, a recent study^52^ of IM program directors showed that many program directors lacked familiarity with the ACGME diversity standard, and among program directors who were aware of the diversity standard, many stated that they lacked the programmatic resources to address issues of diversity, equity, and inclusion in their program. Last, our cohort included 2 IM residency classes, and it is unknown whether these racial and ethnic disparities in assessments persisted in subsequent cohorts. Additionally, because of incomplete GME Track data, we excluded nearly 10% of residents from our original study cohort. Nevertheless, our study involved a national cohort of IM residents and, to our knowledge, is the largest investigation of racial and ethnic equity in GME performance assessments to date.

## Conclusions

In this cohort study, Asian and URiM IM residents received lower Milestone ratings than their White peers, especially in the PGY2 of GME training, which may reflect bias in assessment. This disparity in assessment may limit opportunities for physicians from minoritized racial and ethnic groups and hinder workforce diversity.

## Notes

Supplement 1. **eFigure 1.** Racial and Ethnic Differences in Milestones Scores in Each of the 6 Competency Domains in the Unadjusted Model **eFigure 2.** Racial and Ethnic Differences in Milestones Scores in Each of the 6 Competency Domains in the Adjusted Model

Supplement 2.
Data Sharing Statement
