# Supplementary material for: Racial and Ethnic Differences in Internal Medicine Residency Assessments
Source: JAMA Netw Open. 2022 Dec 29;5(12):e2247649. doi: 10.1001/jamanetworkopen.2022.47649 (PMC9857126; doi:10.1001/jamanetworkopen.2022.47649)
Supplement: Supplement 1. — eFigure 1. Racial and Ethnic Differences in Milestones Scores in Each of the 6 Competency Domains in the Unadjusted Model eFigure 2. Racial and Ethnic Differences in Milestones Scores in Each of the 6 Competency Domains in the Adjusted Model [file jamanetwopen-e2247649-s001.pdf]

Supplementary Online Content

Boatright D, Anderson N, Kim JG, et al. Racial and ethnic differences in internal medicine residency assessments. *JAMA Netw Open*. 2022;5(12):e2247649. doi:10.1001/jamanetworkopen.2022.47649

eFigure 1. Racial and Ethnic Differences in Milestones Scores in Each of the 6 Competency Domains in the Unadjusted Model

eFigure 2. Racial and Ethnic Differences in Milestones Scores in Each of the 6 Competency Domains in the Adjusted Model

This supplementary material has been provided by the authors to give readers additional information about their work.

© 2022 Boatright D et al. *JAMA Network Open.*

eFigure 1. Racial and Ethnic Differences in Milestones Scores in Each of the 6 Competency Domains in the Unadjusted Model

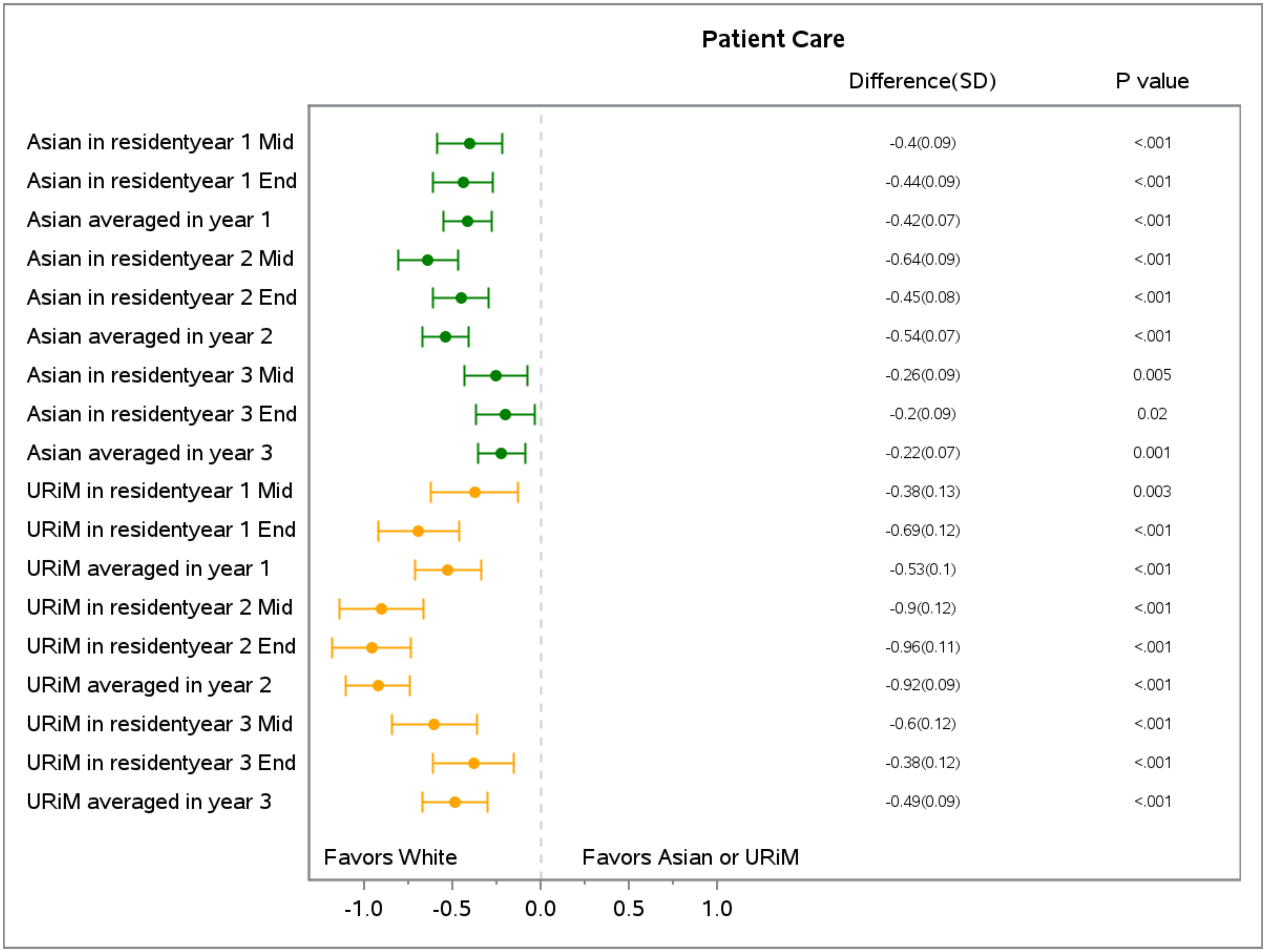

eFigure 1a. unadjusted Patient Care

© 2022 Boatright D et al. *JAMA Network Open.*

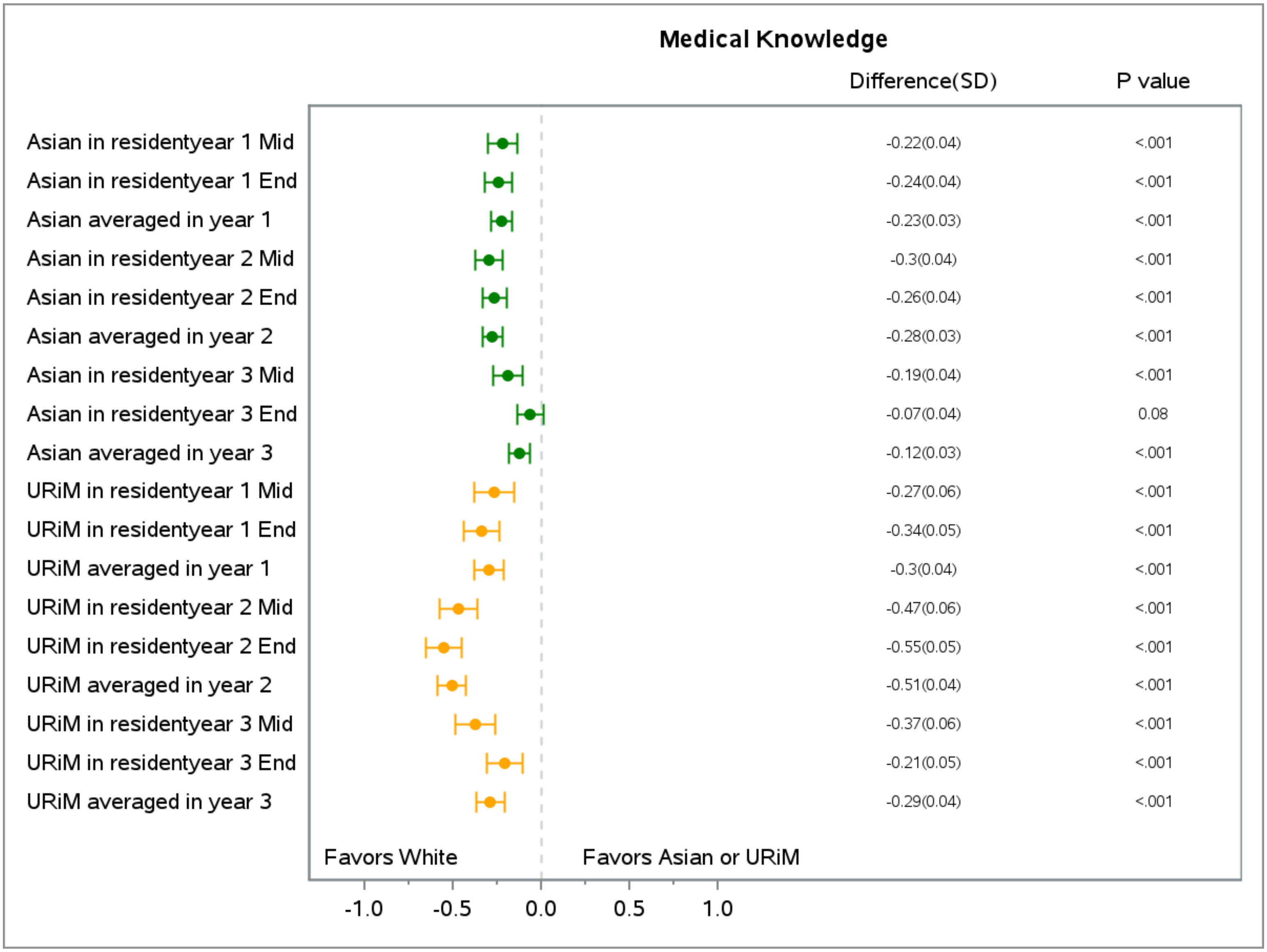

eFigure 1b. unadjusted Medical Knowledge

© 2022 Boatright D et al. *JAMA Network Open.*

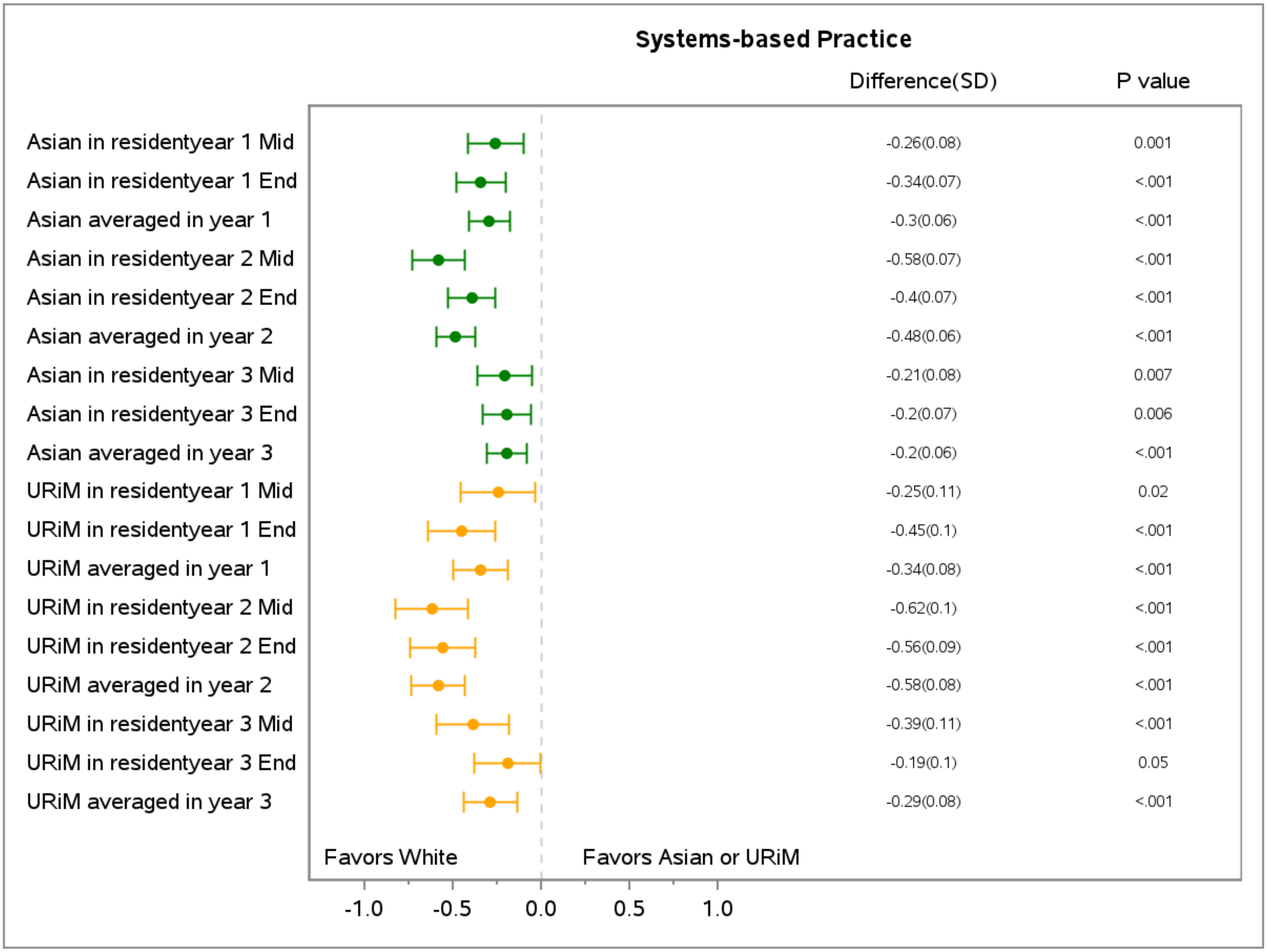

eFigure 1c. unadjusted System-based Practice

© 2022 Boatright D et al. *JAMA Network Open.*

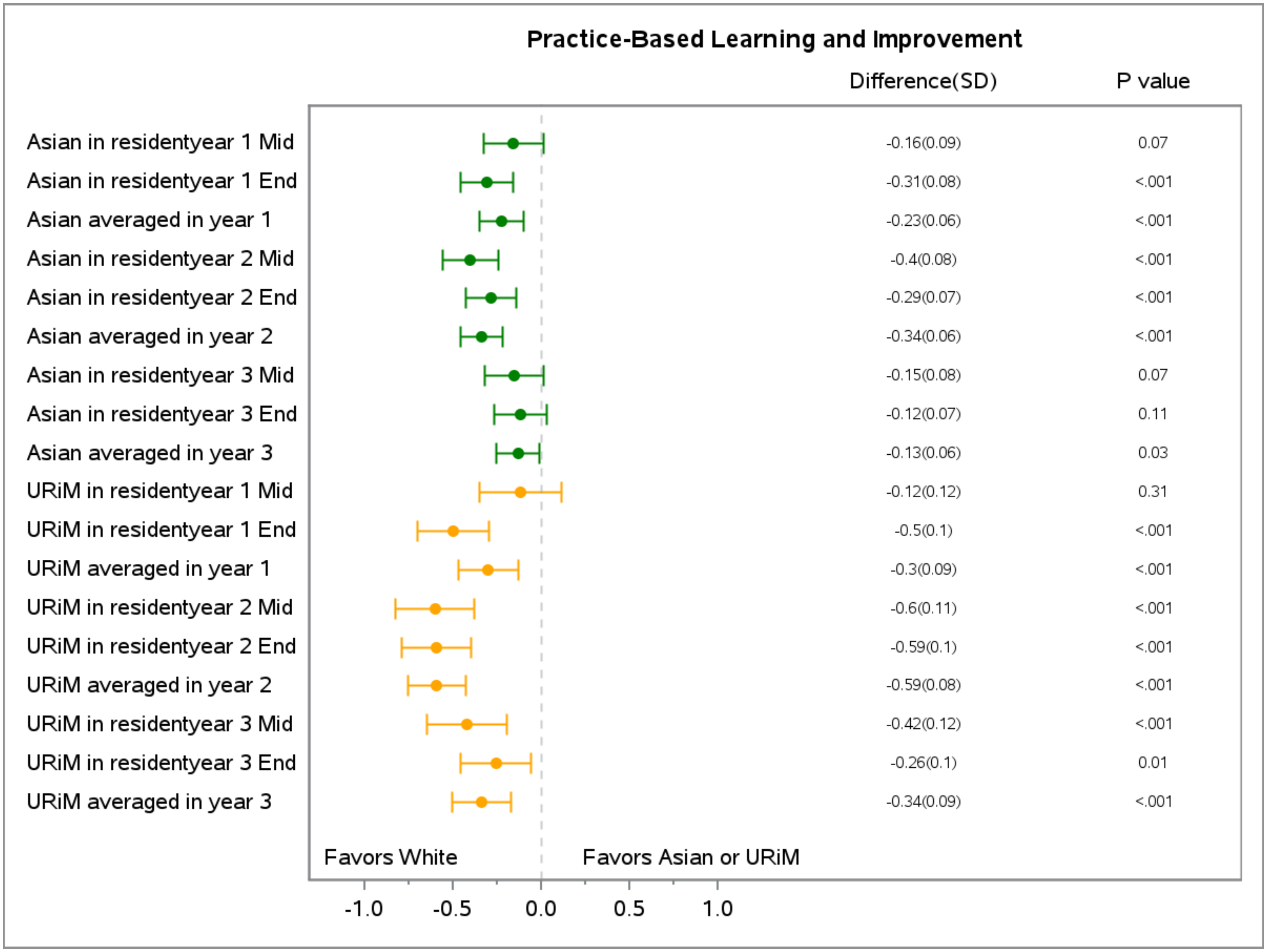

eFigure 1d. unadjusted Practice-based Learning and Improvement

© 2022 Boatright D et al. *JAMA Network Open.*

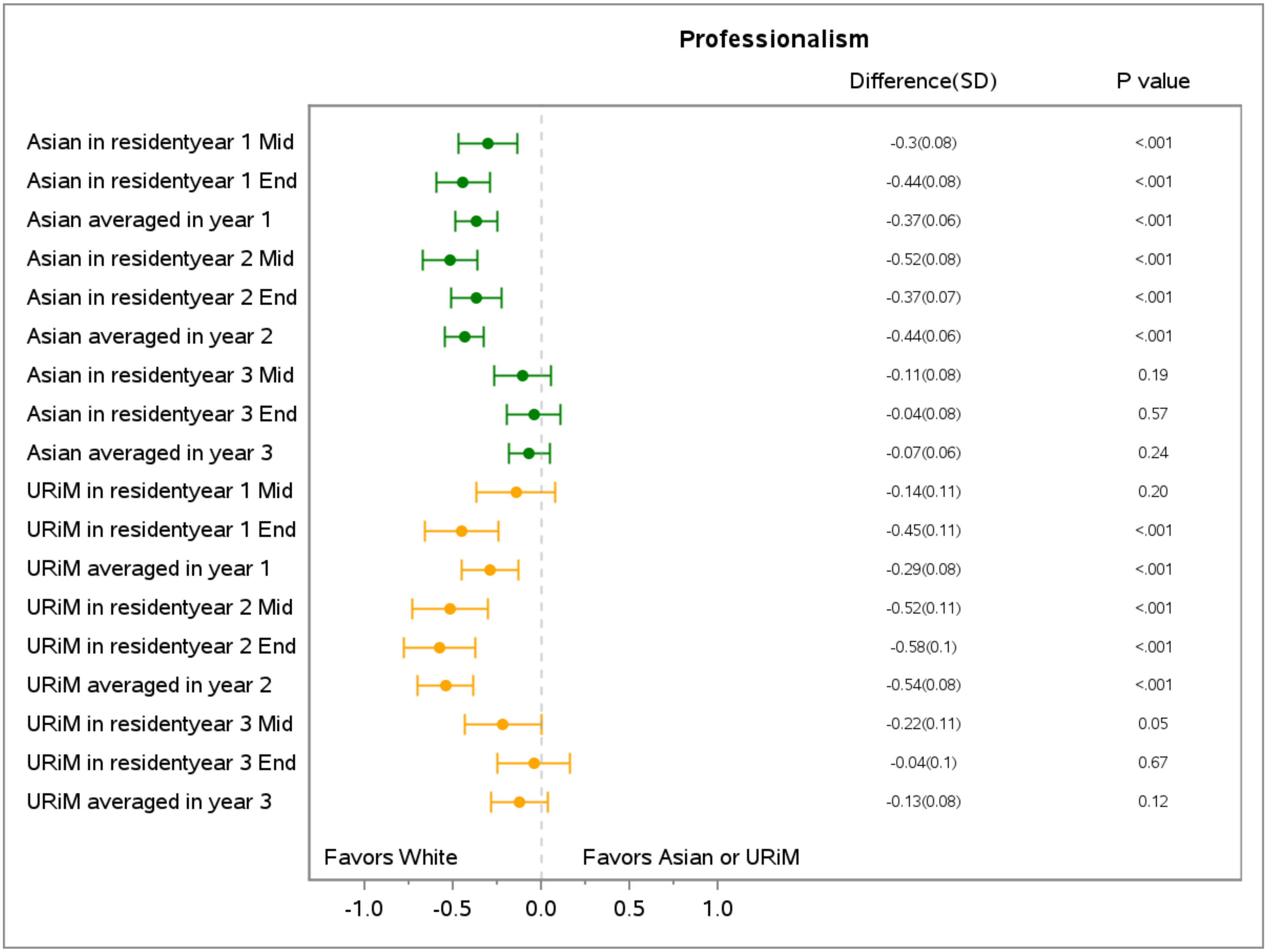

eFigure 1e. unadjusted Professionalism

© 2022 Boatright D et al. *JAMA Network Open.*

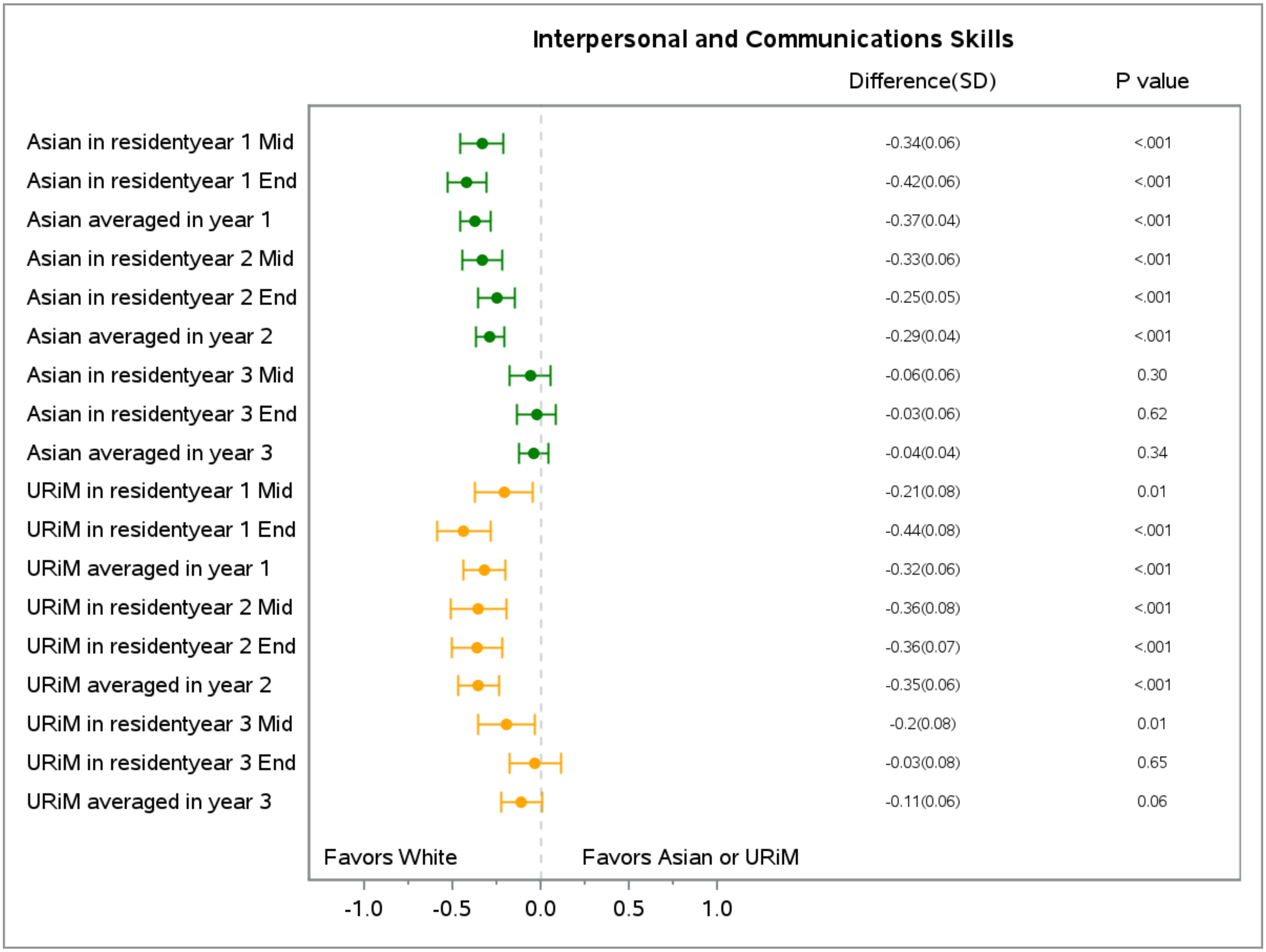

eFigure 1f. unadjusted Interpersonal and Communications Skills

© 2022 Boatright D et al. *JAMA Network Open.*

eFigure 2. Racial and Ethnic Differences in Milestones Scores in Each of the 6 Competency Domains in the Adjusted Model

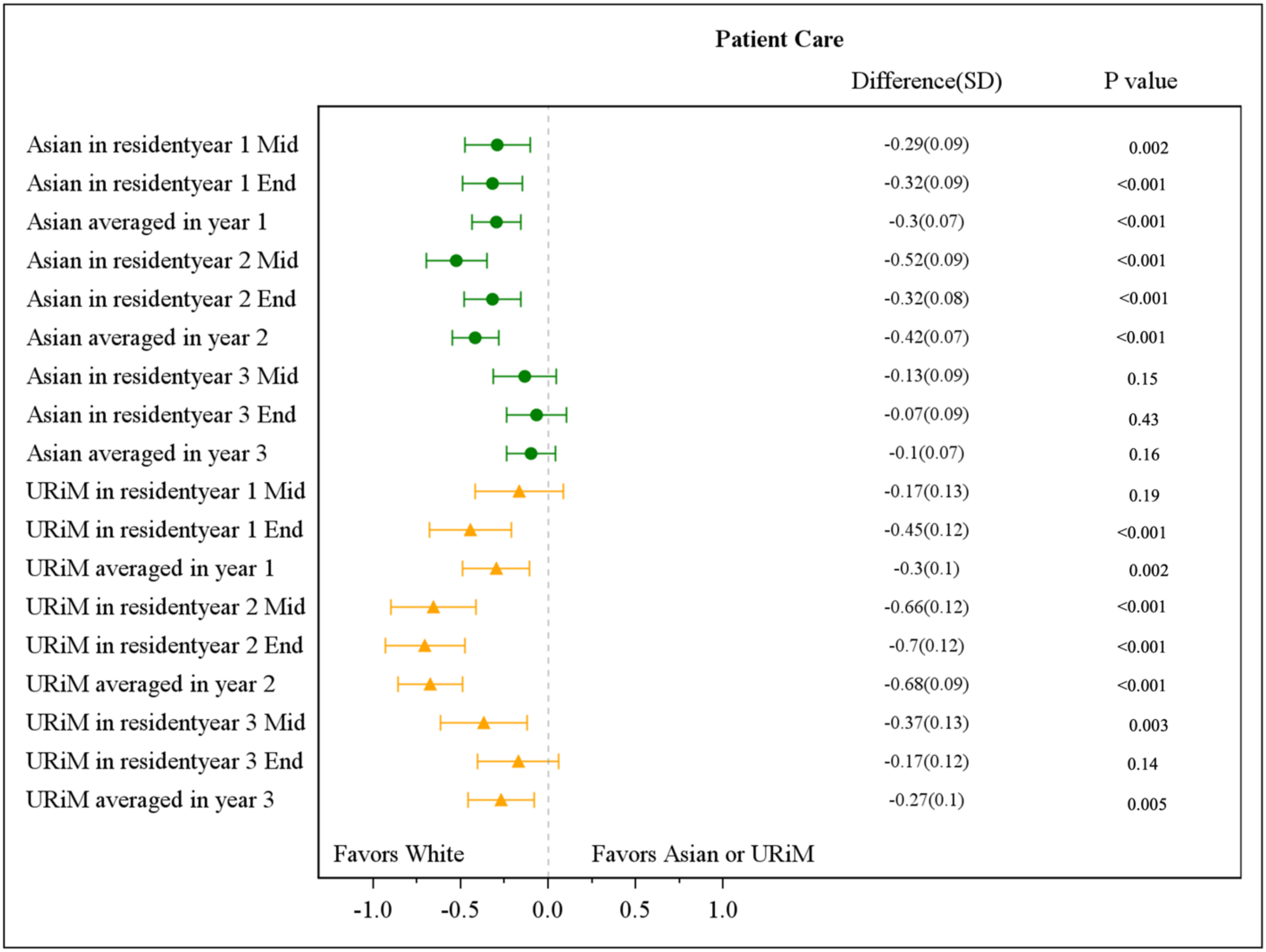

eFigure 2a. adjusted Patient Care

© 2022 Boatright D et al. *JAMA Network Open.*

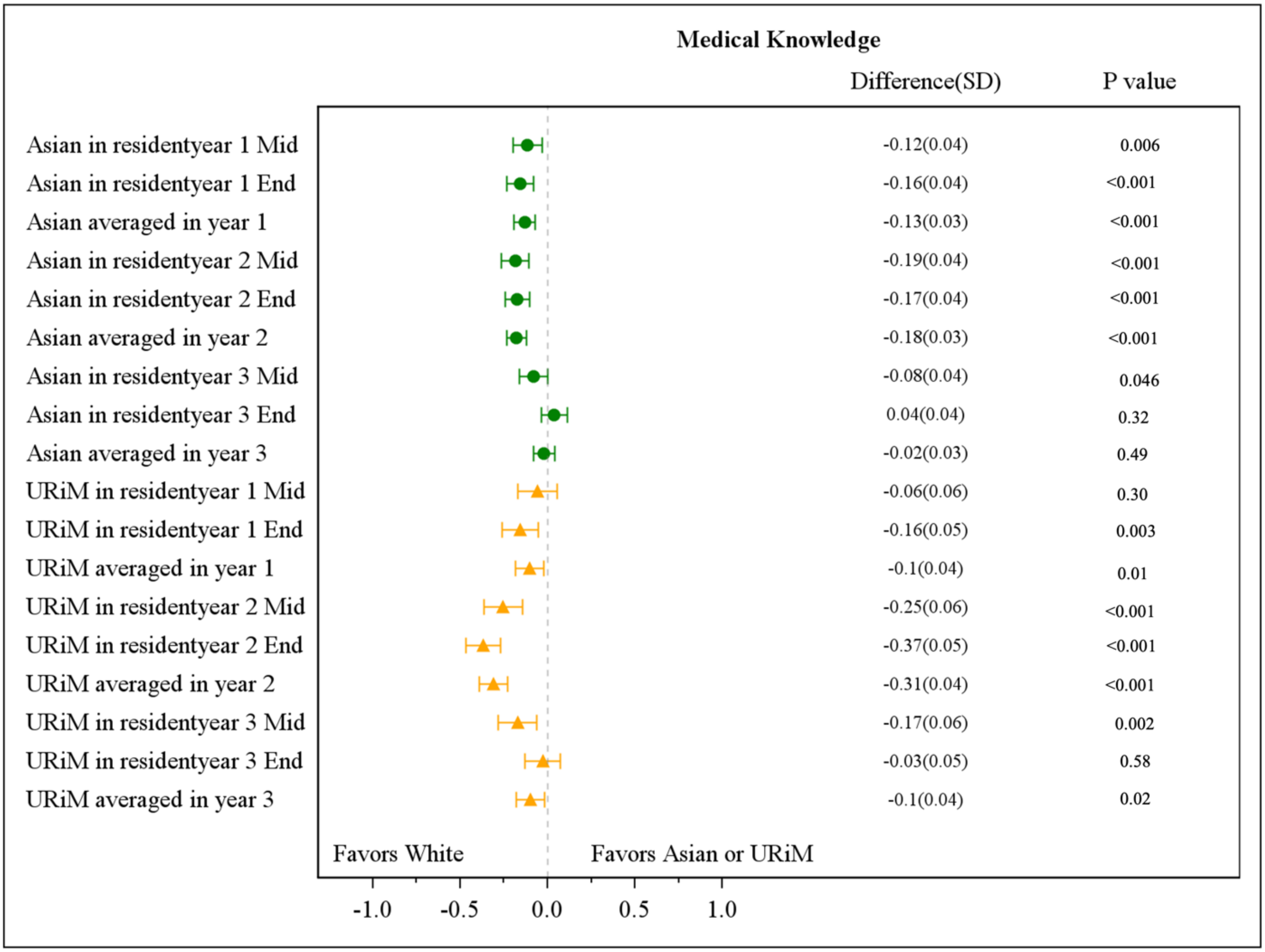

eFigure 2b. adjusted Medical Knowledge

© 2022 Boatright D et al. *JAMA Network Open.*

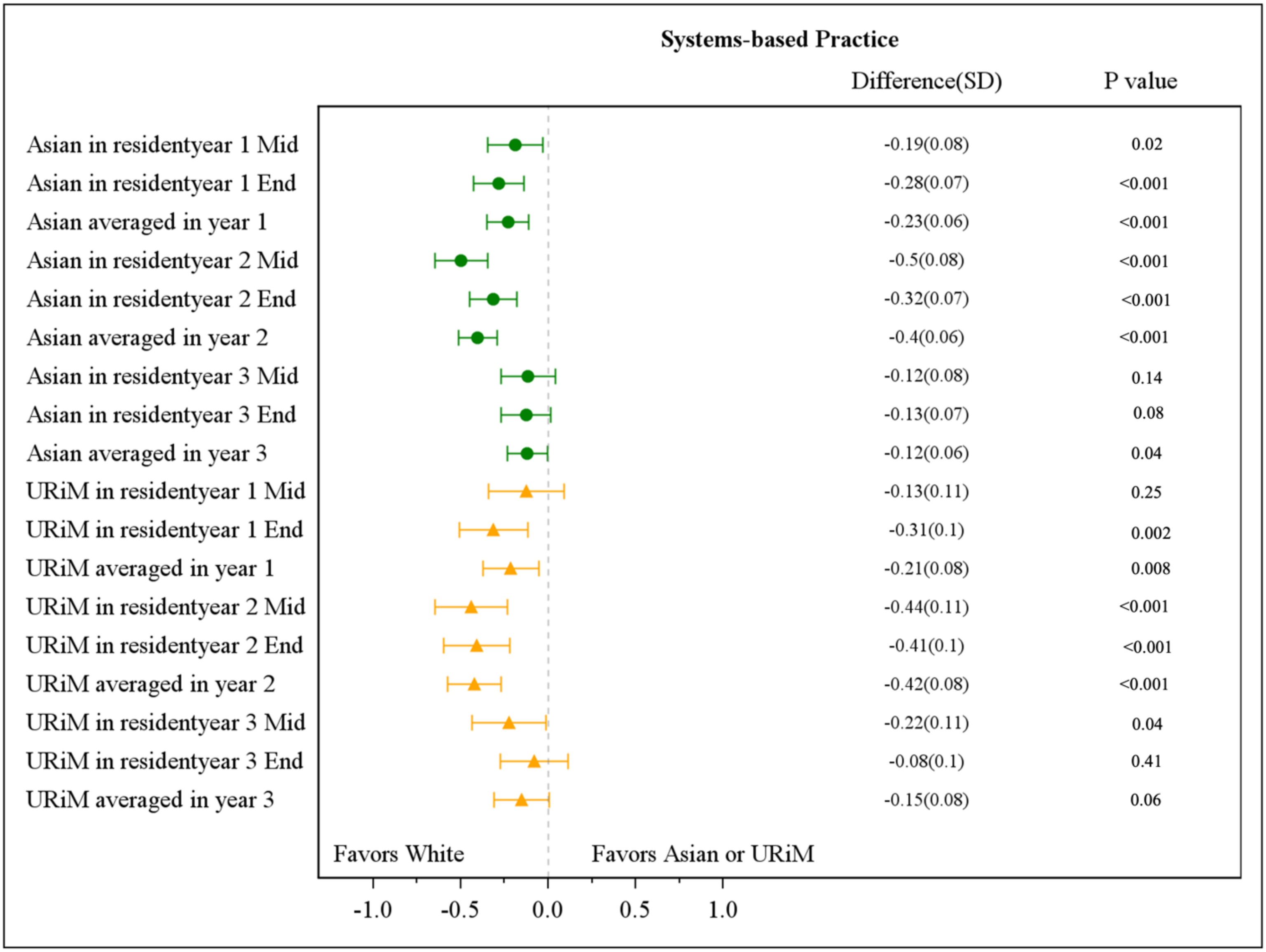

eFigure 2c. adjusted System-based Practice

© 2022 Boatright D et al. *JAMA Network Open.*

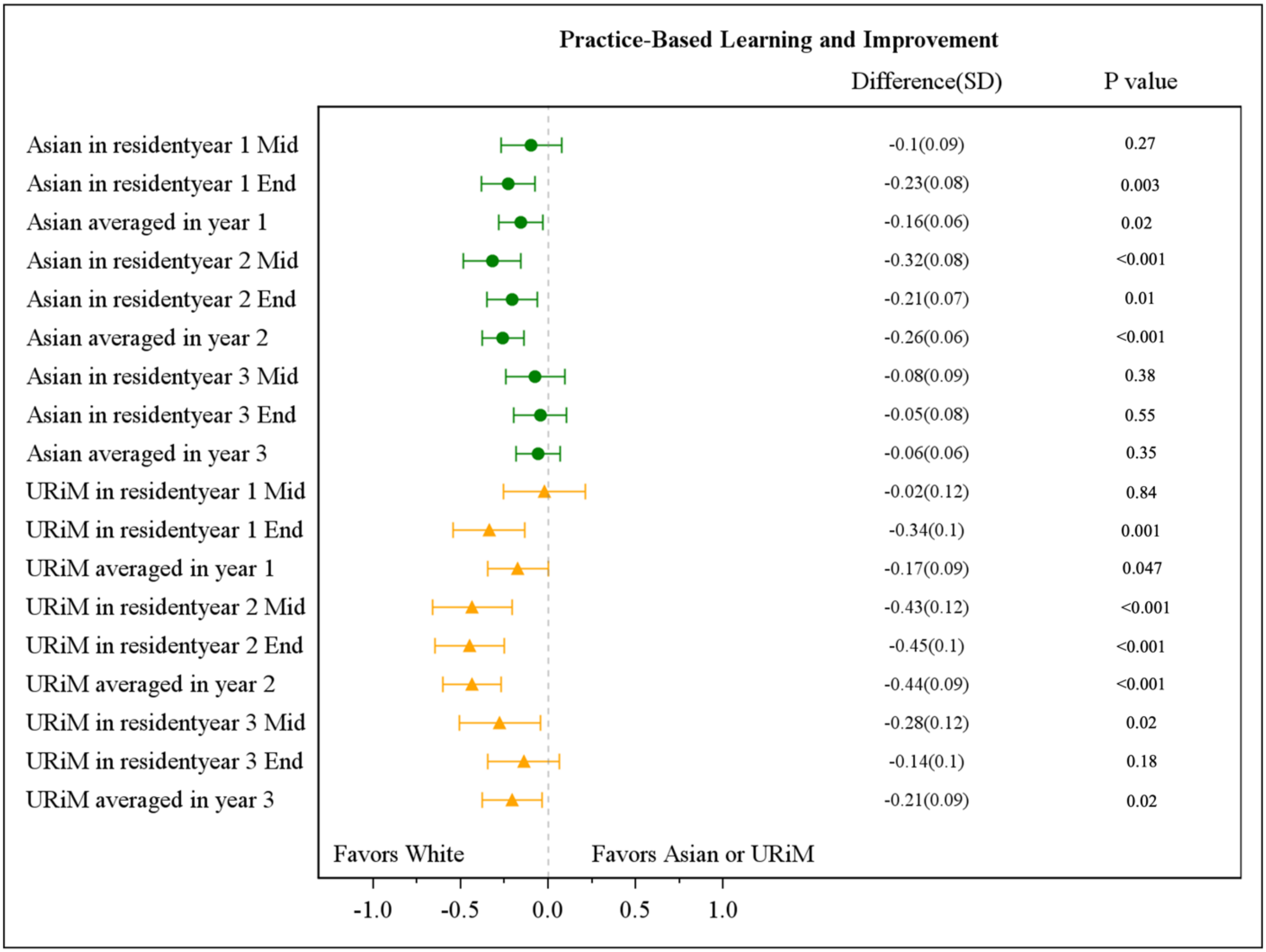

eFigure 2d. adjusted Practice-based Learning and Improvement

© 2022 Boatright D et al. *JAMA Network Open.*

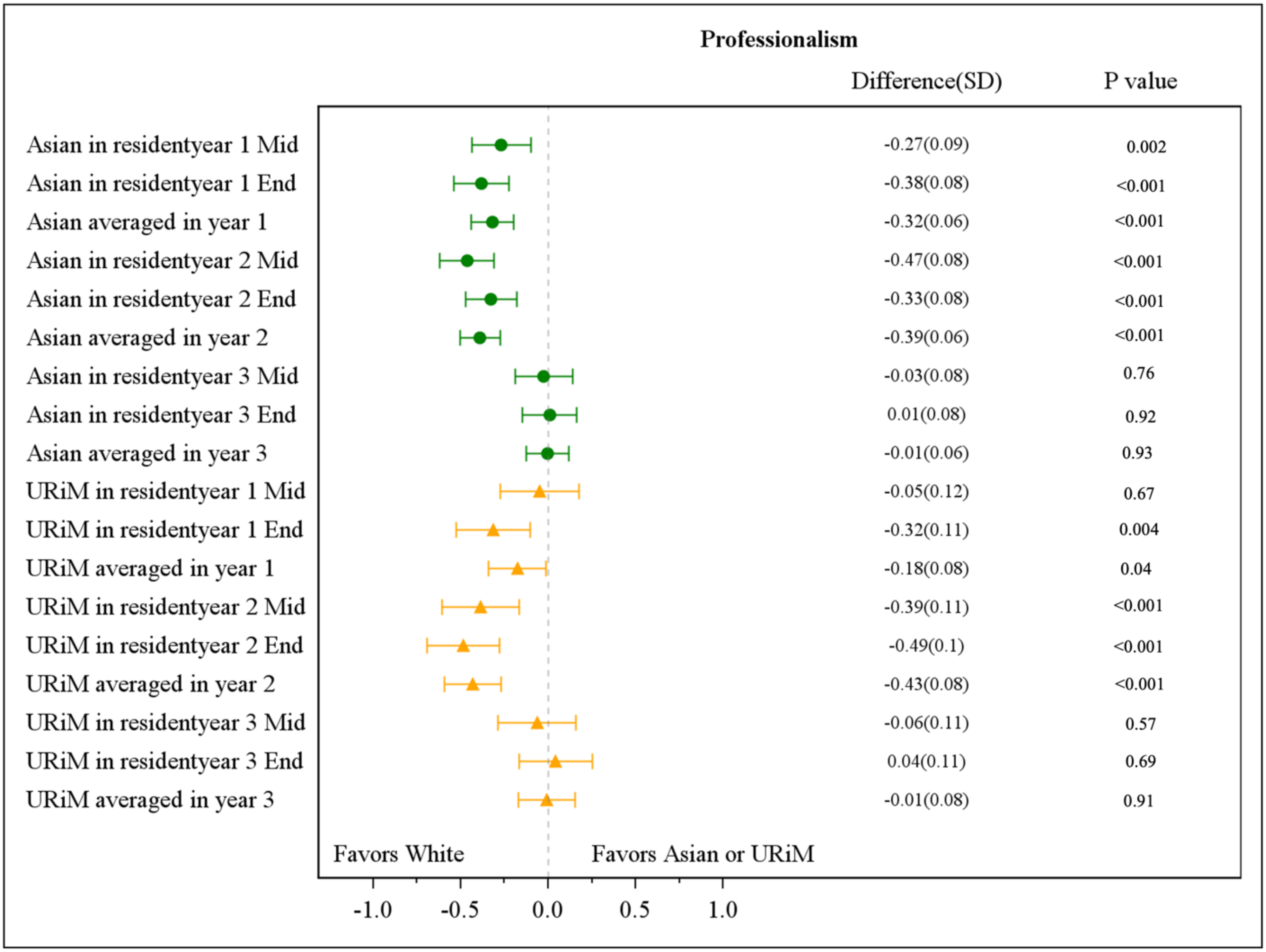

eFigure 2e. adjusted Professionalism

© 2022 Boatright D et al. *JAMA Network Open.*

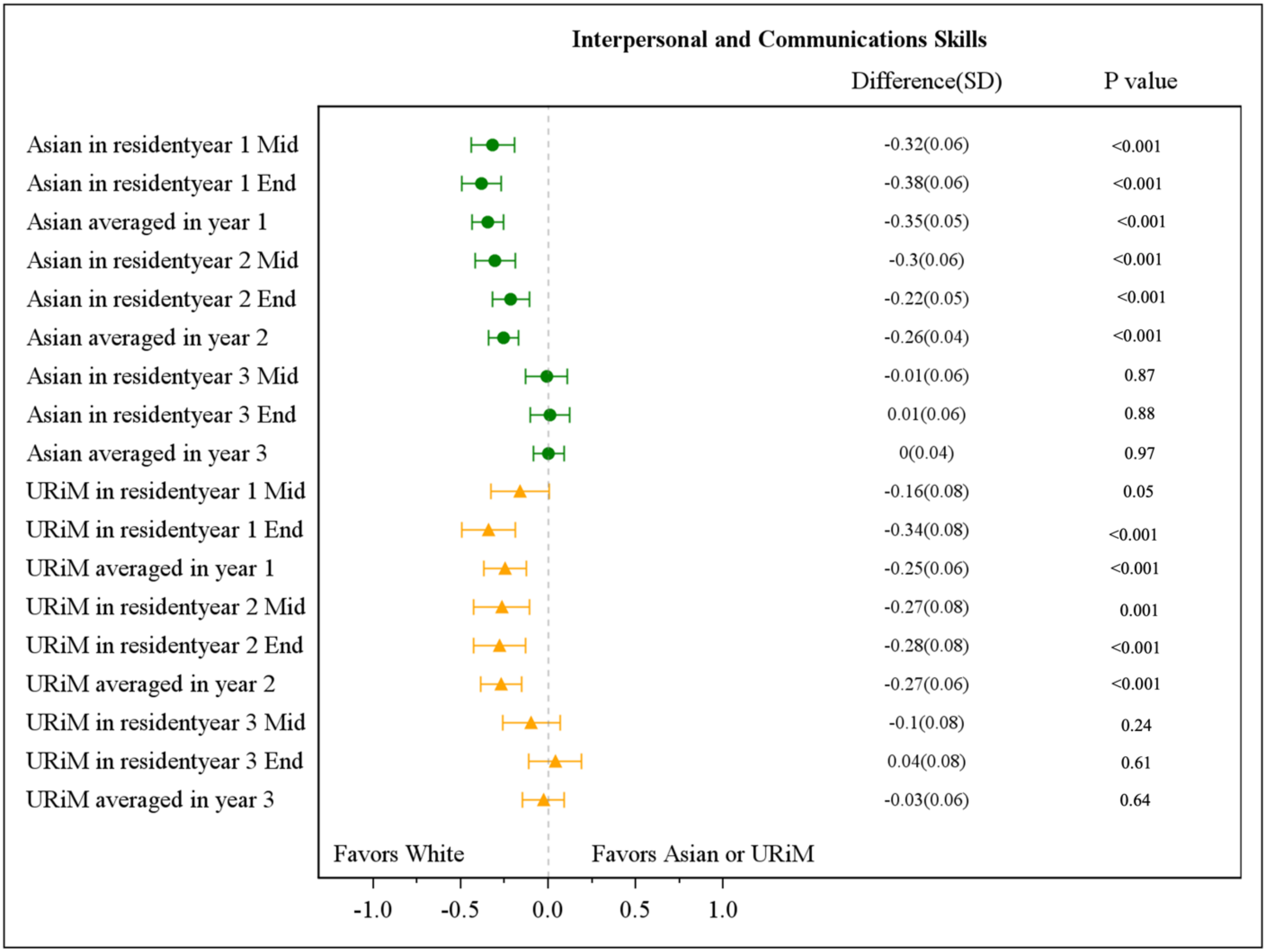

eFigure 2f. adjusted Interpersonal and Communications Skills

© 2022 Boatright D et al. *JAMA Network Open.*
