# Supplementary material for: Racial and Ethnic Differences in Internal Medicine Residency Assessments
Source: JAMA Netw Open. 2022 Dec 29;5(12):e2247649. doi: 10.1001/jamanetworkopen.2022.47649 (PMC9857126; doi:10.1001/jamanetworkopen.2022.47649)
Supplement: Supplement 2. — Data Sharing Statement [file jamanetwopen-e2247649-s002.pdf]

Data Sharing Statement

Boatright. Racial and Ethnic Differences in Internal Medicine Residency Assessments. *JAMA Netw Open*. Published December 29, 2022. doi:10.1001/jamanetworkopen.2022.47649

Data
Data available: No
